# Fire fighters as basic life support responders: A study of successful implementation

**DOI:** 10.1186/1757-7241-17-16

**Published:** 2009-04-02

**Authors:** Christian Bjerre Høyer, Erika Frischknecht Christensen

**Affiliations:** 1 Aarhus Fire Department. Ny Munkegade 15, City of Aarhus, DK-8000 Aarhus C, Denmark; 2 Department of Anaesthesiology, Aarhus Sygehus, University Hospital of Aarhus, Noerrebrogade 44, 8000 Aarhus C, Denmark & Central Denmark Region

## Abstract

**Background:**

First responders are recommended as a supplement to the Emergency Medical Services (EMS) in order to achieve early defibrillation. Practical and organisational aspects are essential when trying to implement new parts in the "Chain of Survival"; areas to address include minimizing dispatch time, ensuring efficient and quick communication, and choosing areas with appropriate driving distances.

The aim of this study was to implement a system using Basic Life Support (BLS) responders equipped with an automatic external defibrillator in an area with relatively short emergency medical services' response times. Success criteria for implementation was defined as arrival of the BLS responders before the EMS, attachment (and use) of the AED, and successful defibrillation.

**Methods:**

This was a prospective observational study from September 1, 2005 to December 31, 2007 (28 months) in the city of Aarhus, Denmark. The BLS responder system was implemented in an area up to three kilometres (driving distance) from the central fire station, encompassing approximately 81,500 inhabitants. The team trained on each shift and response times were reduced by choice of area and by sending the alarm directly to the fire brigade dispatcher.

**Results:**

The BLS responders had 1076 patient contacts. The median response time was 3.5 minutes (25^th ^percentile 2.75, 75^th ^percentile 4.25). The BLS responders arrived before EMS in 789 of the 1076 patient contacts (73%). Cardiac arrest was diagnosed in 53 cases, the AED was attached in 29 cases, and a shockable rhythm was detected in nine cases. Eight were defibrillated using an AED. Seven of the eight obtained return of spontaneous circulation (ROSC). Six of the seven obtaining ROSC survived more than 30 days.

**Conclusion:**

In this study, the implementation of BLS responders may have resulted in successful resuscitations. On basis of the close corporation between all participants in the chain of survival this project contributed to the first link: short response time and trained personnel to ensure early defibrillation.

## Background

In cardiac arrest the time to defibrillation is of major importance for survival. One way of achieving early defibrillation is by using Basic Life Support (BLS) responders: lay persons trained to perform BLS (chest compressions and mouth-to-mouth ventilation) and use an automatic external defibrillator (AED) (which is able to distinguish between shockable and non-shockable cardiac rhythms and advise the BLS responder to defibrillate or not) [1]. Guidelines 2000 for Cardiopulmonary Resuscitation [2,3] recommends the use of BLS responders such as fire fighters trained in BLS and the use of AED as a Class IIa recommendation [1]. Studies show that the use of BLS responders is not always as successful as it might be expected to be [4-12]. Increase in survival among victims of out-of-hospital cardiac arrest (OCHA) has been found with the addition of BLS responders to the existing Emergency Medical Services (EMS) [4-9], while others find only modest or no improvements in cardiac arrest survival with the use of BLS responders [10,11]. One controlled clinical trial has shown a 21% relative increase in the proportion of cases reached within 8 minutes and a 33% relative increase in survival to hospital discharge after implementation of a BLS responder programme [6]. Another study, a controlled cohort trial, found an unchanged hospital discharge survival rate after implementation of a police-manned BLS responder system to the existing fire department-based BLS responders and EMS [11].

The lack of effect of BLS-responders might be due to weaknesses in organizational issues and implementation. van Alem et al. observed in a prospective randomized trial of BLS responders in Amsterdam a median time interval between collapse and first shock of 668 seconds and that their intervention only caused a decrease in time to first defibrillation of 101 seconds [10]. This was partly explained by delays in communication between the emergency medical system and first responder dispatch centres. The OPALS II study reported a mean response "call receipt to vehicle stops with defibrillator" time of 318 seconds in the intervention phase.

As increasingly emphasised, these studies reflect that science alone is not enough. Training and implementation are crucial for gaining the benefit of science in cardiac arrest – expressed as "The formula of survival" [13,14]. In Denmark, BLS responders are only scarcely used, and there are no published reports on successful resuscitation by out-of-hospital BLS responders, except a casuistic report from an airport [15]. The lack of experience in Denmark and the findings by van Alem et al. inspired to do a study with the focus on implementation of BLS responders. Our aim was to implement a system using BLS responders to attend cardiac arrests and situations with a high risk of cardiac arrest in an urban area with relatively short EMS response time by focussing on the following elements of implementation: 1) minimizing dispatch time by short decision-making about activation of BLS responders 2) efficient and quick communication 3) area with appropriate driving distances to allow for defibrillation within five minutes, 4) training of BLS responders and 5) feed-back to BLS responders. Success criteria for implementation was defined as arrival of the BLS responders before the EMS, attachment (and use) of the AED and successful defibrillation.

## Methods

This was a prospective observational study from September 1, 2005 to December 31, 2007 (28 months). Patient data (identity, symptoms, etc.) were only collected when the BLS responders arrived before the EMS.

### Setting

**Figure 1:**
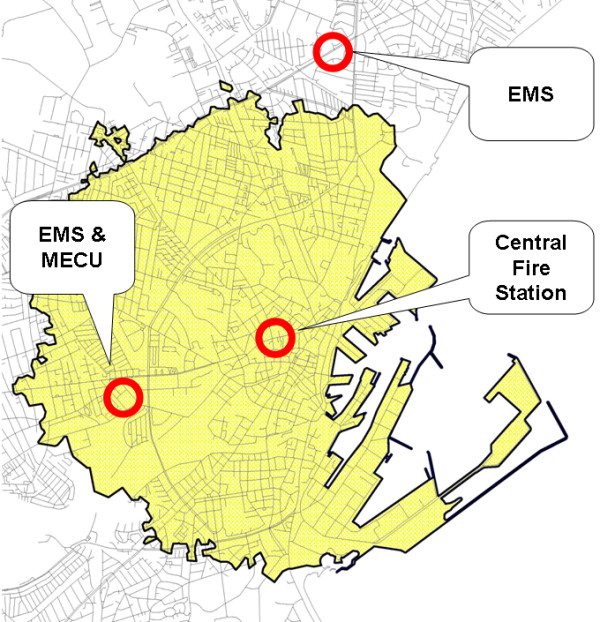
**Area**. The area covered by the BLS responders was defined as all addresses within three kilometres (driving distance) from the Central Fire Station (the coloured area). The area is urban and includes most of the industrial harbour (a minor part of the piers are further than 3 kilometres away). The third EMS-station is located outside the illustration. Abbreviations: BLS Basic Life Support, EMS: Emergency Medical Services, MECU: Mobile Emergency Care Unit.

The city of Aarhus is mainly urban with 330,000 inhabitants. Using the definition of early defibrillation ("shock delivery within 5 minutes from EMS call receipt" [1]) the coverage of the BLS responders was defined to be an area within three kilometres driving distance from the main fire station (figure 1). The population in this area was approximately 81,500 throughout the study period. The central fire station is located in the centre of Aarhus with road distances to the ambulance stations at 2.1, 3.8, and 7.8 kilometres (Figure 1). The EMS is a two tier system with ambulances manned with a paramedic and an emergency medical technician and a Mobile Emergency Care Unit (MECU) manned by an anaesthesiologist and a paramedic trained to assist the physician. The MECU is dispatched if life threatening situations are expected or if an ambulance needs assistance (rendez-vous with the ambulances) [16]. Response times (reception of dispatch message-to-arrival) for ambulances and the MECU averaged 6.7 and 9 minutes, respectively, in 2003 (most recent numbers prior to initiation of the study). Other statistical measures were not obtainable. Electronic dispatch messages were sent simultaneously to the relevant services (EMS, police, or fire department).

In order to improve dispatch we defined criteria were defined for dispatch of the BLS responders as an attempt to improve the positive predictive value. The criteria (all to be met in the dispatch message) were defined as 1) condition described as "cardiac disease", or "'unspecified" AND "unconscious", 2) MECU requested, 3) age >8 or unknown, and 4) BLS responders available. Evaluation of messages and decision about dispatch of the BLS responders was solely performed by the Operations Control Centre at Aarhus Fire Department. Predefined electronic templates ensured proper geographical distribution automatically. Dispatch messages within the defined area were simultaneously sent to the Operations Control Centre and the EMS service. The EMS dispatch procedure was otherwise unchanged.

### Time measurement and registration on-scene

Dispatch requests to the fire department are registered electronically with information about type, address, and time among others. Time for departure and arrival of fire department vehicles are registered electronically in the same database. During this study, patient records were to be filled in upon dispatch. Patient records included information about departure and arrival, patient identity and location, symptoms and treatment, as well as actions performed before the arrival of the BLS responders. The AED stores cardiac rhythm as well as events (voice prompts, defibrillation), and sound. Response times for the BLS responders were calculated from fire departments' dispatch database and from the patient records in case information about arrival was missing for technical reasons. Response time is defined as the interval between reception of the dispatch message at Aarhus Fire Department and arrival of the BLS responders on scene. There is no single registration system, let alone database, combining information from the different dispatch centres (The National Emergency Call Centre, the EMS and the Fire Department). The only way to determine if the BLS responders arrived before or after the EMS was manual review of all patient records filled in by the BLS responders. If the AED was used for defibrillation, the AED recordings were archived for later review by the BLS responders under supervision by a physician (author CBH).

### Training

Prior to this study all fire fighters were certified in Basic Life Support (12 hour course). Additional training (a total of 8 hours) in the use of AED and ethics, personal safety, and cooperation with the EMS was mandatory. On each shift, two fire fighters were assigned as BLS responders and obliged to re-train BLS and use of AED during the shift. Training sessions were recorded via the mannequin used and were routinely reviewed by a physician (author CBH), who also supervised and debriefed the fire fighters. During the study period, ERC Guidelines 2000 were followed in all aspects, including recommendations for diagnostics, treatment, and safety measures [1,17].

### Equipment

A single, small fire vehicle (VW Sharan) with first aid equipment and an AED (Medtronic Physio-Control Lifepak 500). The AED was chosen due to electrode compatibility with defibrillators used in all ambulances in our area (Medtronic Physio-Control Lifepak 12), thus avoiding delay in treatment caused by changing the self-adhesive defibrillation pads.

### Statistics

Estimation of time interval between the arrival of the BLS responders and the EMS was done using the AED recordings of sound and cardiac rhythm in cases when the defibrillator was used. Time intervals are stated in minutes:seconds with 25^th ^and 75^th ^percentiles.

### Ethics and permissions

Permission from The Central Denmark Region Committees on Biomedical Research Ethics was not necessary according to Danish law (correspondence 2004-2.0/16, March 22 2004). Data was stored according to Danish law and permission was obtained from the Danish Data Protection Agency (application 2005-52-3088/14.14P20).

## Results

**Figure 2:**
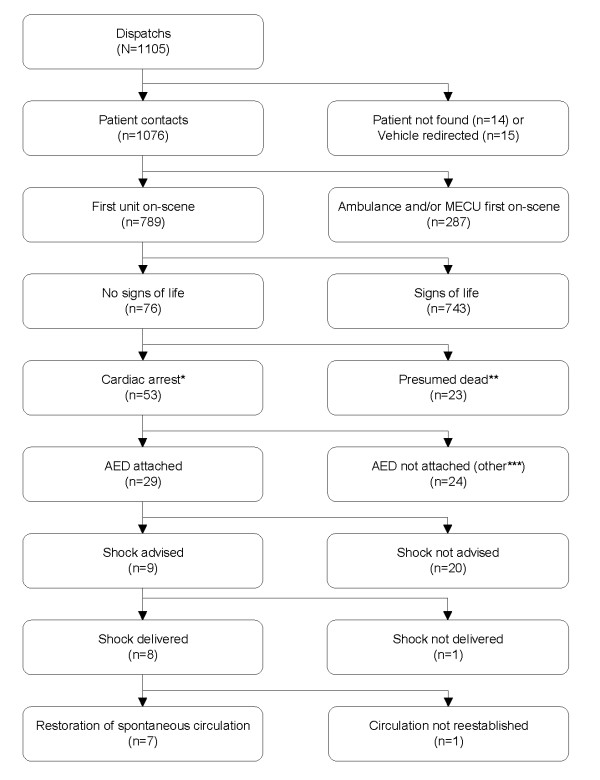
**Flowchart of patients**. * Cardiac arrest: patient unresponsive and with abnormal or no breathing but not presumed dead due to post-mortem rigor and livor mortis. ** Post-mortem rigor and post-mortem livor present. *** Due to arrival of ambulance and/or Mobile Emergency Care Unit before the automatic external defibrillator could be attached, for example.

The total number of alarms was 1105 (1.4 day^-1^) and eight patients were defibrillated (flowchart in Figure 2). There was no patient contact in 29 cases, leaving 1076 dispatches evaluated in the following. In 81.9% of all dispatches (881/1076) the response time could be calculated using electronically stored values. In 156 cases (14.5%) calculation was done by inclusion of values from the patient records. Thus, response time was known in 96.4% of dispatches (1037/1076). The median response time in these 1037 cases was 3.5 minutes (25^th^/75^th ^percentiles 2.8; 4.3 minutes, respectively). In nine cases response time was longer than 10 minutes due to imprecise address or difficulties in locating the patient in locations as factory plants or shopping centres. The BLS responders arrived before the EMS in 73.3% of all dispatches (789/1076). Cardiac arrest was found in 53 cases and the AED attached 29 times (54.7%). A shockable rhythm was diagnosed in nine patients, and eight were defibrillated using the BLS responders' AED; the ninth was defibrillated with the EMS' defibrillator. Restoration of spontaneous circulation (ROSC) was obtained by defibrillation of the BLS responders in seven cases, in the eighth case ROSC was not established until treatment at hospital. Of the seven persons obtaining ROSC on-scene six survived more than 30 days, the seventh died after two days. The interval between AED attachment and the arrival of EMS was estimated from the AED recordings and ranged from 0.5 to 6 minutes.

## Discussion

This study showed it possible to successfully implement a BLS responder unit in an urban area with arrival before the ambulance in 73% cases, attachment of the AED in 55% of cardiac arrests and defibrillation in eight out of nine patients with shockable rhythm, of whom seven gained ROSC and six survived more than 30 days.

The important goal was not response time per se, but the sequence of arrival. Our second aim was if the BLS response unit actually did attach the AED and furthermore if this would result in defibrillations. One of the reasons why the AED was only attached in 55% was that the EMS arrived only shortly later than the BLS responders, before the BLS responders attached the electrodes to the patient.

A study on the use of police as BLS responders included the finding that an existing fire department-based BLS responder unit arrived first in sequence, not only before the police, but also before the existing EMS: In average, the fire departments' BLS responders arrived 3.8 and 4.9 minutes before the EMS [11]. In another study it was shown, that fire fighters as BLS responders arrived in average 1.3 minutes before the EMS leading to the conclusion that fire fighters have time enough to initiate lifesaving interventions [18]. These figures are not directly comparable with our study, but we find them in line with our results: the BLS responders arrived before the EMS in approximately three out of four cases.

Studies regarding the use of first responders in urban areas have shown a variety of results. The OPALS II study showed an increase in survival from OHCA attributed to several factors, including the use of BLS responders [6]. Conversely, van Alem et al. found delays in decision-making and communication to reduce the potential benefits of BLS responders. In our study, the median response time (from receipt of dispatch message to arrival of vehicle) was 210 seconds.

### Considerations about implementation

Implementation of BLS responder systems must be weighed carefully against local factors, and what organisational measures can be done to improve the system

We chose specifically an area small enough to give low response times. By this we have shown it possible to implement a BLS responder system with success. However, at the same time, this makes it difficult to generalize results to other areas, as they may differ significantly from the area in this study. Further strengthening of the dispatch system, such as using health care professionals in the dispatch system, might increase the specificity of the dispatches. Finally, BLS responders do not do it alone: The chain of survival includes other links, and the survival of each patient depends as much on these: Continued BLS in the ambulances, advanced life support by the physician from the MECU, and advanced and intensive care upon arrival to the hospital.

### Dispatch

The lack of a central collection of information from all authorities involved in the response to victims of cardiac arrest is a weakness in this study. A consequence is, that we can not obtain the actual response times from initial call to arrival on-scene. Another is that while we can quantify overtriage, we have no possibility to quantify undertriage, as we can not obtain the total number of cardiac arrests within a certain area. Cardiac arrest was found in only 4.9% of all patient contacts (53/1076, Figure 2) showing a massive overtriage of the BLS responders. This was expected as it has been described in two studies from our area: One found dispatch to acute coronary syndrome to have a positive predictive value of only 45% [16], the other a positive predictive value of only 39% when comparing persons classified as 'unconscious' in the dispatch message to the Glasgow Coma Scale-score upon arrival of the MECU at scene [19]. Emergency calls in Denmark are directed to the National Emergency Call Centre operated by the police (non-health care personnel) which may contribute to both over- and undertriage. Kuisma et al. found a median time from call receipt to dispatch of a BLS response unit to be 77.1 seconds [20]. A similar value is not obtainable in Denmark. The time used by the police-operator from initial call to sending a dispatch message is only available as an overall average for all emergency calls (including theft, domestic disturbances, and alike).

In 2008 this overall average was 94 seconds [21], but due to the nature of calls the time to send dispatch messages to the EMS may be much shorter. A receipt is sent electronically from the EMS/fire departments when dispatch message is read; overall average for this is 12 seconds [21]. The response time for the BLS responders, from receipt of dispatch message at the fire department, could be calculated in most of the cases (96.4%); in the majority of these the BLS responders arrived in less than five minutes (75^th ^centile 4.25 minutes). The remaining 3.6% missing values can be explained by the personnel's "intention-to-treat"-approach leading to a lack of registration. Quantification of differences in response time was not possible as no single database contains this information.

### Training

Throughout the study period, the fire fighters followed the resuscitation guidelines from year 2000. The guidelines from year 2005 were not available in Danish: The Danish version of the ERC Guidelines 2005 was not published until late April 2007 when the Danish Resuscitation Council released a pamphlet about Advanced Life Support on their webpage and in print [22]. Guidelines for BLS and use of AED were not published in the Journal of the Danish Medical Association until November 2008 [23]. Furthermore, the manufacturer of the used defibrillator could not supply software following the new guidelines until after the project finished. This prohibited the use of the newest guidelines which constitutes a weakness in the study regarding the specific treatment of the patients. However, as this paper is about the implementation of the BLS responder system, it does not influence the findings presented.

The experiences of the effect of training on the quality of performance in the real world is differing: Lerner et al. [24] has quoted Sayre (as personal communication) that attachment rate was lower than 5% in spite of bi-annual retraining and quarterly observation and feedback to each police officer, while another study has concluded that high performance in training correlates to high performance in real life [25]. In our study fire fighters were obliged to train BLS and the use of AED on a manikin each time they were on duty as BLS responders, which we believe is a major factor to the high rate of attachment of the AED.

## Conclusion

In summary, we introduced BLS responders in the treatment of OHCA by using existing resources in a favourable geographical setting with approximately 80,000 inhabitants. The BLS responders arrived before the EMS in three out of four times. Eight patients were defibrillated and seven survived to hospital discharge during the study period of 28 months. Our study indicates a need for establishing more accurate dispatch criteria and future studies may address how methods are developed to identify areas in which the implementation of use of BLS responders may be favourable

## Abbreviations

AED: Automatic External Defibrillator; BLS: Basic Life Support; EMS: Emergency Medical Services; MECU: Medical Emergency Care Unit; OHCA: Out-of-Hospital Cardiac Arrest; ROSC: Return Of Spontaneous Circulation

## Competing interests

Author CBH has been working as fire fighter, squad leader and instructor at the Fire Department of Aarhus.

## Authors' contributions

CBH participated in the design, preparations, implementation, training, collection and processing of data, and drafting of the manuscript. EFC participated in the design, preparations, implementation, and drafting of the manuscript. Both authors have read and approved the final manuscript.
